# Experiences and Perceptions of Doctors, Nurses, and Midwives Providing Abortion Care in Australia: A Scoping Review

**DOI:** 10.1111/ajo.70124

**Published:** 2026-05-05

**Authors:** Katie Edmondson, Caroline De Costa, Heather A.Grimes, Cate Nagle

**Affiliations:** ^1^ College of Healthcare Sciences James Cook University Townsville Queensland Australia; ^2^ The Cairns Institute (TCI) James Cook University Cairns Queensland Australia; ^3^ Nursing and Midwifery, College of Healthcare Sciences James Cook University Townsville Queensland Australia

**Keywords:** abortion, Australia, induced, midwifery, nurses, physicians

## Abstract

**Background:**

Doctors, nurses, and midwives are key providers of abortion care for women in Australia. Providing abortion care is a challenging, contentious, and sometimes perilous occupation. Recent changes in the Australian abortion care landscape mean that it is essential to consider the experiences and perceptions of the abortion workforce and to identify future needs.

**Aims:**

The aim of this review was to collate and report the experiences and perceptions of doctors, nurses, and midwives who provide abortion care in Australia.

**Materials and Methods:**

A scoping review was utilised to address this question, applying Arksey and O'Malley's framework with enhancements by Levac et al. Research question development and reporting approaches were informed by Joanna Briggs Institute (JBI) Health.

**Results:**

In total, 19 articles were included in the review. Studies were predominantly qualitative in nature and encompassed all three clinical groups. Most studies included participants from multiple Australian sites, with five focusing specifically on Victorian participants. Overarching themes included: clinical care provision; person‐centred care; social and system‐based factors; provider education, training and networks; abortion and the law; ethical challenges; emotional responses; abortion stigma; and conscientious objection.

**Discussion:**

The experiences of Australian abortion providers reflect shared challenges relating to: training access; ethical and emotional impacts; abortion stigma; and conscientious objection. Navigating changing system, regulatory, and legal frameworks compounds this complexity.

**Conclusion:**

Further research into the experiences of abortion providers will inform support interventions. Legislators, regulators, and health service executives must listen to the needs of providers to ensure service sustainability into the future.

## 1 Background

Doctors, nurses, and midwives who provide abortion care (abortion providers) experience a unique set of challenges in supporting the reproductive choices of their clients. Many factors may impact negatively upon clinicians who provide abortion care, potentially diminishing job satisfaction and reducing the long‐term viability of abortion service provision [1, 2, 3, 4]. These factors may relate to workplace and service delivery issues, clinician and client emotional responses, and broader societal and legislative influences or barriers [1, 2, 3, 4, 5, 6, 7]. The degree to which these challenges are acknowledged and addressed can have a direct impact on clients and the clinicians providing care [6]. These challenges are likely all experienced by Australian abortion providers and are potentially enhanced by additional pressures specific to the Australian setting. Understanding the various factors that may impact Australian abortion providers is essential.

In Australia, abortion care has traditionally been provided primarily as a surgical service in private facilities [8]. Recent changes in access to the abortion medications mifepristone and misoprostol have led to an expansion of services provided by community‐based general practitioners (GPs) [8, 9]. Publicly funded health services are also playing an increasing role in later surgical and medical abortion care [9, 10, 11, 12]. Therapeutic Goods Administration (TGA) amendments in 2023 removed restrictions confining MS‐2‐Step prescribing to medical practitioners, permitting other registered health professionals to provide this abortion medication [13]. This, paired with recent legislative amendments in Queensland, Western Australia, and Victoria now supports nurses and midwives in those states to provide early medical abortion (EMA) care within their scopes of practice [14, 15, 16]. Essentially, a broader group of healthcare providers in Australia is providing abortion care in increasingly diverse settings.

Legislative factors unique to the Australian setting have also impacted abortion care providers over the last 20 years [7, 17]. Each Australian jurisdiction has transitioned from a criminalised to a decriminalised framework, through the introduction of specific laws safeguarding legal abortion in each state and territory [7, 17]. The Australian Capital Territory (ACT) and Victoria were among the earliest jurisdictions to decriminalise abortion, enacting the *Medical Practitioners (Maternal Health) Amendment Act 2002* and the *Abortion Law Reform Act 2008* respectively [17]. Other states and territories have taken longer to introduce specific legislation, with Queensland, New South Wales (NSW), Northern Territory (NT), South Australia (SA), and Western Australia (WA) all enacting their legislation to decriminalise abortion between the years of 2018 and 2024 [18, 19]. It is important to consider the impacts on clinicians who must embed these new legislative frameworks into both public and private practice.

Broader societal factors and stigma can also influence providers' experience of performing abortion care. The practical intent of abortion laws often reflects current political and social ideology [7, 17], the effects of which are also felt by abortion providers. Martin et al. [20] found that public and private beliefs about abortion can be both polarised and emotive, resulting in both the abortion recipient and the provider being subjected to stigmatisation and abuse. Stigmatisation of this nature can extend to physical threats in polarised settings, creating genuine safety risks [1, 2, 5]. The impact of these factors can be significant for abortion providers, both on a personal level and within the workplace [1, 4].

Workplace‐based factors have been observed to directly undermine abortion providers' capacity to practice. In their survey of abortion providers in southern United States of America (USA), Chowdhary et al. [1] recognised that when clinicians feel stigmatised or alienated within their workplace, they are likely to experience high levels of stress and are at risk of burnout. Sorhaindo and Lavelanet [5] built on these concerns, noting that if workplaces are ill‐equipped for abortion care provision in general, clinicians will feel unable to provide the quality of care they believe is indicated. Ultimately, workplace factors have great potential to influence whether staff perceive their abortion care provision as positive or negative.

Conscientious objection can negatively impact the experiences of abortion providers. The Australian Medical Association defines conscientious objection as a situation where “a doctor, as a result of a conflict with his or her own personal beliefs or values, refuses to provide, or participate in, a legal, legitimate treatment or procedure which would be deemed medically appropriate in the circumstances under professional standards” [21]. Abortion‐related conscientious objection can be informally applied by a range of clinicians [4, 22], or legislated for specific scenarios [7, 23]. Sometimes it takes the form of denial of service and blocking of onward referral; however, these activities are not supported by the conscientious objection provisions in legislation [7]. Abortion providers working in settings with many conscientious objectors often find themselves feeling overburdened and demoralised [2, 4].

The potential emotional impact sustained by health professionals when delivering abortion care must be considered [2, 3, 4]. Performing or assisting with specific abortion procedures may cause distress for clinicians [4, 24]. Additionally, some abortion clients had intensive psychosocial support needs which staff may find challenging [4]. Shimoyama and Tsukamoto [25] surveyed nurses in Japan and found that those who experience ongoing vicarious trauma from providing abortion were at risk of compassion fatigue and burnout. The sustainability of services is at risk if the psychological needs of abortion providers are not addressed, and they feel less able to deliver abortion care [2, 3, 4].

## 2 Aims

Examining what is known about Australian abortion providers' experiences when delivering abortion care is essential to designing strategies that increase workforce sustainability in Australia. Abortion providers in Australia are well‐positioned as key informants for this purpose. The aim of this review was to collate and report the experiences and perceptions of doctors, nurses, and midwives who provide abortion care in Australia.

For the purposes of this review, abortion is defined as the intentional “removal of pregnancy tissue or the fetus and placenta from the uterus” [26]. This term is interchangeable with ‘termination of pregnancy’ or “ToP”. Additionally, while abortion seekers are referred to as ‘women’ in this review, as this is the terminology used in the reviewed articles, the authors acknowledge and include all pregnant people who seek abortion care.

## 3 Materials and Methods

A scoping review was identified as the appropriate method for this project as the intent was to examine the extent, range, and nature of the literature available describing Australian abortion providers' experiences and perceptions [27]. A broad lens was indicated as it was expected that there may be a wide variation in the types, nature, and context of research available on this topic [28]. The scoping review methodology would also support the identification of gaps in the literature warranting further exploration [27].

Using the Population, Concept, Context (PCC) framework [29] the question was developed by the authors, whereby the ‘population’ identified was doctors, nurses, and midwives who are the primary abortion providers in Australia. The ‘concept’ for exploration was their experiences and perceptions of providing abortion care, and Australia was the agreed ‘context’.

The review followed Arksey and O'Malley's [27] framework, enhanced by recommendations from Levac et al. [30] and Joanna Briggs Institute (JBI) Health [29, 31, 32]. The five key steps were: identifying the research question; identifying relevant studies; study selection; charting the data and collating; summarising and reporting the results [27]. Levac et al.'s [30] enhancements involved clarifying and linking the research question to the purpose of the review, ensuring feasibility by balancing the search strategy with the need to be comprehensive, and applying an iterative approach to study selection and data extraction. The question and reporting approach were informed by JBI Health [29, 31]. Reporting for this scoping review was consistent with the Preferred Reporting Items for Systematic reviews and Meta‐Analyses (PRISMA) checklist for scoping reviews [33].

All studies exploring the experiences and perceptions of doctors, nurses, and midwives (or a combination) providing direct abortion care in Australia, either currently or in the past, were included. No restriction was applied with respect to gestation at abortion or care setting. Only peer‐reviewed, original research studies were included in the analysis, with no restriction on the type of research methodology utilised. No limits were placed on date of publication nor language. Reviews, editorials, letters to the editor, and grey literature were excluded from selection. Any article without accessible full text was also excluded. The reference lists from full text articles screened were searched for additional references.

The PCC framework components were used to identify keywords required to develop the literature search strategy [29]. To minimise risk that a relevant article might be missed, and to ensure a systematic approach was applied, separate searches were conducted in five bibliographic databases, including MEDLINE (OVID), CINAHL Complete, PsychINFO (Proquest), Emcare (OVID), and Scopus. The search terms applied were developed in collaboration with a librarian expert in systematic literature searches and tailored to the specific search strategies and terms appropriate to each database.

**FIGURE 1:**
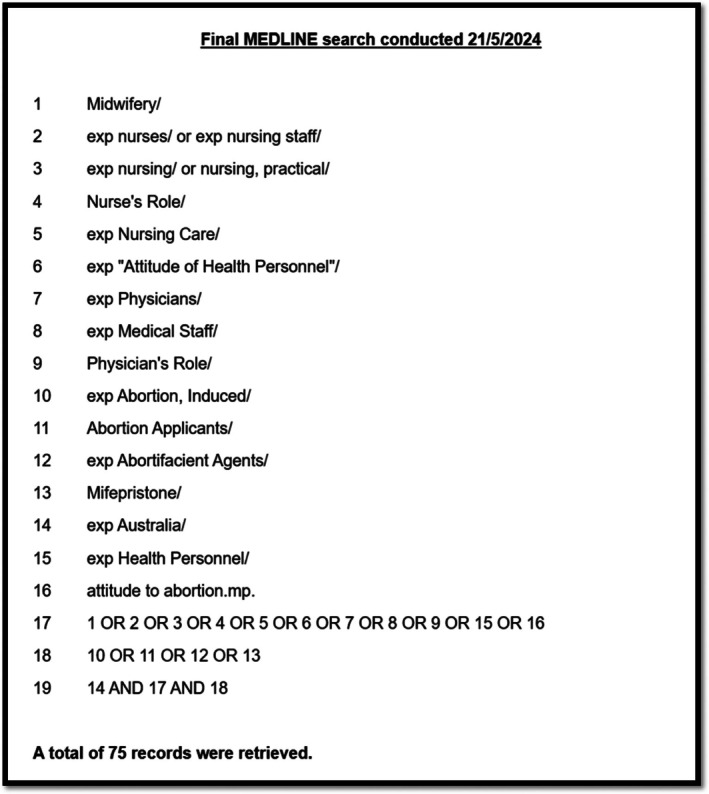
MEDLINE search strategy.

The MEDLINE (OVID), CINAHL Complete, and Emcare (OVID) searches were conducted using keywords, and all associated Medical Subject Headings (MeSH) headings were mapped for inclusion. Key PCC components were connected using Boolean phrases to create an integrated search strategy which was developed in these three databases. The keywords and associated MeSH headings already identified were then collated into search strings for each PCC component. These search strings were utilised in combination with Boolean operators ‘AND’ and ‘OR’ to search the PsycINFO (Proquest) and Scopus databases. The final search strategy for the MEDLINE (OVID) database is provided as an example in Figure 1. All final searches were undertaken on 21/5/2024 and records identified were exported into an EndNote database. Duplicate records were removed.

Title and abstract screens were conducted for all articles by K.E. and replicated by C.N. and H.A.G. to confirm agreement on articles included in the next stage of screening. A total of 34 articles were then reviewed by K.E. for full‐text screening. Full‐text reviews were replicated by another member of the research team to test agreement.

Several of the articles screened were produced by one of the research team members. To avoid a conflict of interest, C.D.C. did not participate in the title and abstract screen. Additionally, any of C.D.C.'s articles considered for full‐text screening were assessed for inclusion by K.E. and either C.N. or H.A.G.

Data extraction was performed using a standardised data extraction form developed specifically for this review. The extraction form was piloted by C.N. and K.E. for two of the articles to confirm reliability. K.E. then performed data extraction and charting on all remaining articles. Findings from the data charting process were tabulated in an Excel spreadsheet. Data extracted included descriptive and thematic elements. General information was collected in relation to the article itself including: the year published; authorship; country; and the publishing journal. The study type, aims/objectives, year, date of data collection, population studied, and sample size were included. The location of the study participants, type of abortion care provided, and setting of abortion care were also identified. Once the data was extracted it was tabulated in a data extraction table in reverse chronological order (Appendix 1). The key findings and themes explored by each article in respect to experiences and perspectives were extracted into an Excel spreadsheet.

Conducting a critical appraisal in a scoping review is contentious as the intent is to map and describe the evidence rather than find a synthesised answer to a specific clinical question [29]. If a critical appraisal were suitable for the subject matter, a systematic review design would have been applied instead. For this reason, the research team members decided not to assess the quality of the included studies.

An exemption to ethics review has been provided for this study by the James Cook University Human Research Ethics Committee, application/reference number 24H‐9662.

## 4 Results

**FIGURE 2:**
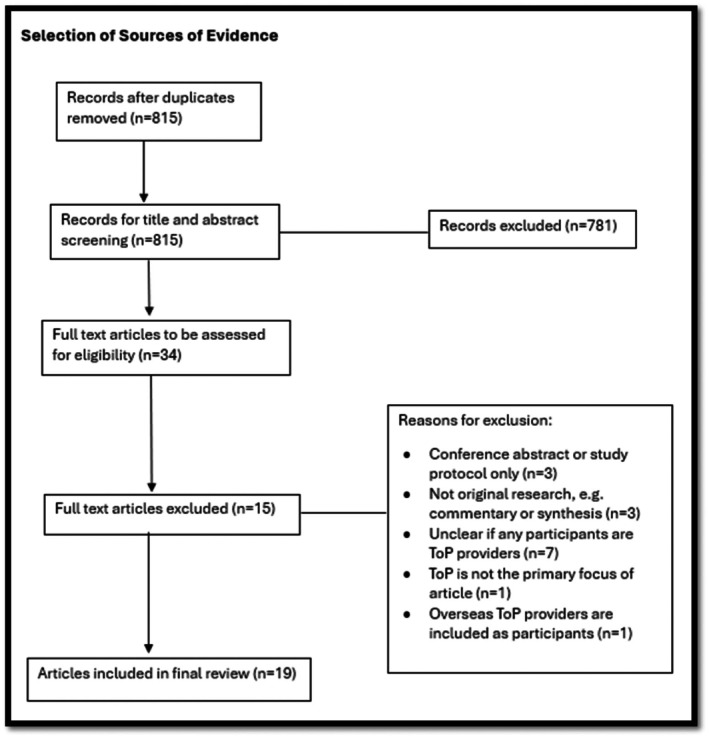
Source selection flowchart.

Initially, a total of 815 citations were identified from searches of electronic databases after duplicates were removed (Figure 2). No additional articles were identified for consideration from the reference lists of articles included in full‐text screening. After the application of the selection criteria, 19 articles were eligible for inclusion in the scoping review.

Appendix 1 presents key descriptive information extracted from 19 articles, including the first author, year of publication, title, Australian state/territory in which participants reside, aims, and stated objectives of the study, the study type (e.g., qualitative interviews, cross‐sectional survey, etc.), participants and sample size. Key findings are also summarised in this table. Nine of the included articles presented separate analyses from the same four studies, meaning that the content examined in this review is sourced from only 10 separate data sets.

Most studies (14/19) were conducted using a qualitative descriptive methodology. Doctors were participants in many studies (*n* = 9), with some focusing on specific medical sub‐specialties. The remaining articles involved nurses and midwives (*n* = 3) or had mixed participants (*n* = 7). While most of the articles focused on data collected from abortion providers across multiple Australian sites (*n* = 9), a significant number focused specifically on Victorian participants (*n* = 5).

**FIGURE 3:**
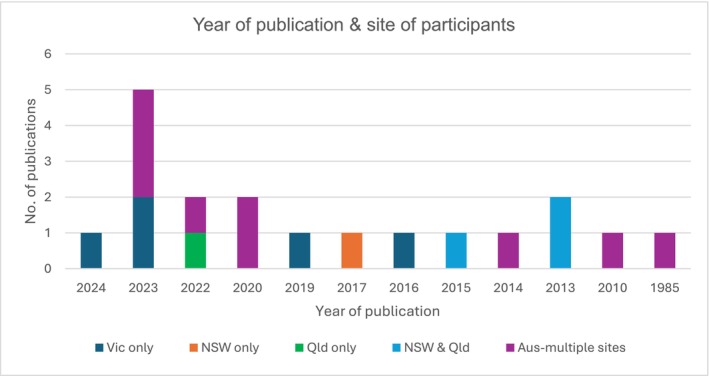
Year of article publication and site of participants.

The included articles were published between 1985 to 2024 (Figure 3); however, the majority (*n* = 15) were published within the last 10 years. Aside from Victoria, the Australian Capital Territory (ACT), and Tasmania, jurisdictions did not decriminalise abortion until after 2017 [18]. Eleven articles were published after 2018, a time of rapid legislative change in several states and territories, and 15 articles were published after MS‐2‐Step for medical abortion was listed by the TGA in 2014 [51].

**TABLE 1:** Thematic summary.

Overarching themes	Articles incorporating theme	Findings
Clinical care provision	[22, 34, 35, 36, 37, 38, 39, 40, 41, 42, 43, 44, 45, 46, 47, 48]	Five articles focused on provider experiences in performing early medical abortion (EMA), while four focused on provision over 20 weeks of gestation [22, 34, 35, 36, 37, 41, 43, 44, 45] EMA providers had variable opinions about the best models of care for abortion provision [41, 43, 44] Care pathways, team skill‐mix and clinical follow‐up were all identified as key issues for many clinicians [35, 39, 41, 43] Clinicians providing abortion over 20 weeks of gestation highlighted the unique nature of the abortion care they provide [34] Several articles identified diverse views on the role of nurses and midwives in abortion provision, [35, 40, 44] Newton et al. [44] found that general practitioners (GPs) had diverse views on whether ‘average’ GPs or specialist clinics should provide abortion. This was reflected by Dawson et al. [43], with some participants believing abortion should be confined to specialised settings Cheng et al. [42] and de Costa et al. [47] found that 91.9% and 88.6% (respectively) of RANZCOG Fellows and trainees surveyed supported provision of abortion in the public setting. Only 69.3% of Queensland sexual health nurses and midwives surveyed in 2022 were supportive of public sector abortion provision [40]
Person‐centred care	[22, 23, 34, 35, 36, 38, 39, 40, 42, 44, 45, 46, 47, 49, 50]	In Malek's [34] exploration of clinicians providing abortion care over 20 weeks of gestation, participants focused on “delivering holistic abortion care that centred women and pregnant people's needs and autonomy” One of Baird's [49] participants recalled practicing in the time of illegal abortion, noting that “it certainly coloured my whole view of the need for abortion to be a safe, sterile, accessible service for women” (p. 426) Singh et al. [35] interviewed General Practitioners (GPs) caring for culturally and linguistically diverse (CALD) women seeking abortion, who highlighted the need for interpreters, gender‐specific providers and cultural capability skills The ability to provide person‐centred care was closely linked to care provider wellbeing [38] Additional complexities were experienced by nurses and midwives providing abortion care to clients experiencing gender‐based violence (GBV), due to safety concerns and the vulnerability of the population [39]
Social and system‐based factors	[22, 23, 34, 35, 36, 37, 38, 39, 41, 42, 43, 45, 46, 47, 49, 50]	de Costa et al. [46] and Black [45] described doctors' frustrations with having to navigate legislative complexities associated with justifying abortion care pre‐decriminalisation Douglas et al. [50] had participants describing the need to “construct an appropriate narrative to justify a termination” (p. 568) Access to care was also noted to be impeded by inadequate service availability, inappropriate services for vulnerable populations, and inadequate staffing or skill mix [38, 39, 44] Dawson et al. [43] and Newton et al. [44] describe the challenges of rural EMA provision and subsequent barriers to access for vulnerable individuals, including First Nations clients Perceived delays to care related to insufficient services, rigid service models and unclear care and referral pathways, as well as conscientious objection caused challenges for providers [23, 36, 41, 43, 44] Two studies focused on the perceived impact of the Victorian TRCs in delaying abortion care over 20 weeks, exploring views around the acceptability, fairness, uniformity, and impacts of these processes [22, 37] Malek et al. [34] referenced the specific impact of the COVID pandemic on delayed service provision and increased demand
Provider education, training and networks	[34, 35, 36, 39, 40, 41, 42, 43, 44, 47]	Clinicians interviewed in some studies indicated that an advanced skill level was required to support abortion provision [38, 39, 40, 43, 44] 92.9% of sexual health nurses (SHN)s and midwives surveyed by Desai et al. [40] believed that abortion should be a part of nursing and midwifery curriculum 97.7% and 90.7% RANZCOG Fellows and trainees interviewed by de Costa et al. [47] and Cheng et al. [42] (respectively) who felt abortion should be part of routine obstetrics and gynaecology practice also believed that abortion practice should be part of their curriculum GPs interviewed by Dawson et al. [43] were positive about existing training but highlighted the need for psychosocial training Participants also noted a preference for robust clinical care networks, including pharmacists and sonographers [43] Deb et al. [41], Newton et al. [44], and Hass et al. [36] all found strong support for mentoring; strong clinical guidance and support, as well as abortion provider networks/communities of practice
Abortion and the law	[23, 37, 45, 46, 49, 50]	Articles conducted prior to abortion being decriminalised identified experiences of complex and restrictive access to abortion after 20 weeks' gestation; the need for a doctor to be manipulative in supporting access to abortion; and the need to construct mental illness justifications for abortion [45, 46, 50] Doctors perceived a genuine risk of criminal charges prior to decriminalisation [45, 46, 50] Keogh et al. [23] explored legislative conscientious objection provisions established post‐decriminalisation which are perceived as both protective and obstructive in different measures The TRCs in Victorian hospitals emerged post‐decriminalisation, with some clinicians perceiving this as being in response to a controversial case referred to the state Coroner for investigation [37] While some participants felt that Victorian TRCs were a reasonable response to the legislative change and reputational risk management, others saw this as organisational gatekeeping [37]
Ethical challenges	[22, 23, 34, 37, 38, 39, 40, 42, 45, 46, 47, 48, 49, 50]	Peterson's [48] study explored abortion providers views on when an abortion would be approved noting maternal ambivalence about abortion (26.5%), reproductive coercion (25.6%) and advanced pregnancy (16.2%) as reasons to discourage an abortion Only 53.5% of SHNs and midwives surveyed by Desai et al. [40] believed that abortion should be offered in any situation Some RANZCOG Fellows and trainees interviewed in 2020 distinguished the justification of abortion for fetal anomaly versus “social” reasons [42]. This was also reflected in de Costa's [47] original survey in 2010 The articles focusing on clinician experiences of Victorian TRCs identified that providers sometimes found abortion decision‐making at later gestations challenging, and welcomed supportive frameworks [22, 37] Mainey et al. [38] highlighted how nurses and midwives working within an unsupportive health system felt forced to actively subvert the system to provide abortion care Doctors providing abortion care before decriminalisation spoke of manipulating the legislative requirements to support an abortion [46, 50]
Emotional responses	[23, 34, 37, 38, 39, 40, 45, 46, 49, 50]	Some participants “commented on the difficulty associated with the dichotomy of caring for women undergoing elective abortion while also attending to women who have had unexpected fetal or neonatal loss” [40] The emotional challenges of providing abortion care at later gestations were reinforced by Malek et al. [34], with participants acknowledging that the situations and procedures involved had the potential to cause staff psychological trauma Nurses and midwives interviewed by Mainey et al. [38] expressed distress elicited by empathy for the woman's situation paired with an inability to facilitate abortion care Providers who were able to secure access to an abortion for a client experienced positive emotional responses [38] Some clinicians expressed a feeling of job satisfaction when they were able to provide much needed abortion care [49] Misuse of legislated conscientious objection provisions, resulting in delays or diminished access to abortion, was found to increase client distress [23]
Abortion stigma	[35, 36, 39, 41, 43, 47, 49]	Some GP EMA providers interviewed by Dawson et al. [43] experienced judgement from colleagues, negative social consequences and were reluctant to promote their abortion services. Doctors in several studies expressed a fear of being perceived as the “abortion doctor” in their practice setting [43, 47] Haas et al. [36] identified abortion stigma as barrier to EMA provision for registered nurses Baird's [49] article recounts an abortion provider's experience receiving hate mail at one work site and fake bomb threats at another Provider safety was identified as a unique risk to clinicians caring for clients experiencing GBV with nurses and midwives who provide this care found themselves threatened by partners [39]
Conscientious objection	[22, 23, 39, 40, 42, 43, 47]	Keogh et al.'s [23] article explores clinician experiences of legislated conscientious objection provisions, noting that they both protected a women's access to abortion, while also being susceptible to manipulation and misuse de Costa et al. [47] and Cheng [42] found that 14.6% and 13.7% (respectively) of RANZCOG Fellows and trainees had religious or conscientious views that would make them totally opposed to providing abortion 7.4% of Queensland SHNs and midwives surveyed in 2022 held views based on religion or conscience that would make them completely opposed to abortion [40] Conscientious objection in healthcare settings was identified as a cause of delayed or limited care for women [22, 39, 43] Dawson et al. [43] noted that the unwillingness of GPs or specific practices to perform abortion care increased the burden on existing abortion providers The ways in which larger institutions conscientiously object to providing abortion care was articulated as a significant barrier to care in several studies [22, 23]

The overarching themes identified from the included articles are present in Table 1. Themes included: clinical care provision; person‐centred care; social and system‐based factors; provider education, training, and networks; abortion and the law; ethical challenges; emotional responses; abortion stigma; and conscientious objection.

Experiences of clinical care were inclusive of the changing methods and settings for abortion provision; models of care; multi‐disciplinary teams, and care pathways. Person‐centred care experiences focused on caring for vulnerable populations and the need for holistic, accessible care models. Social and system‐based experiences related to the: legal and system‐based regulatory frameworks; remote and regional settings; and gestational age at time of abortion request. Participants also gave both positive and negative experiences of abortion provider education and training options while expressing the importance of strong clinician support networks.

Clinician experiences navigating the legal elements of abortion provision, both pre‐decriminalisation and in the context of new abortion‐specific legislation, featured in many studies. Additionally, some clinicians identified ethical challenges when providing care that related to the gestation and indication for abortion, as well as clinician behaviour in relation to abortion requests. Both positive and negative emotional responses to providing abortion care were shared, often emerging in response to other themes identified. Abortion stigma had a clear negative impact on some providers. Finally, managing conscientious objection, either informal or legislated, was a major challenge experienced by clinicians in several studies.

## 5 Discussion

This scoping review provides a comprehensive account of the different experiences and perceptions held by clinicians involved in abortion care in Australia. Despite the breadth and complexity of abortion provider experience, it is noteworthy how few Australian studies were available for review. This may reflect the very recent emergence of abortion as an acceptable topic for academic research and publication in Australia, which has only occurred in the 21st century [17]. The articles included in this review paint an evolving picture of abortion provider experiences in Australia.

Experiences of clinical care provision were described primarily in relation to: abortion care methods; setting of clinical care; and clinician roles. The number of articles focusing on the experiences of GPs providing EMA under 9 weeks of gestation [35, 36, 41, 43, 44] increased significantly after the approval of MS2Step in 2013 [9]. Clinician support for publicly funded abortion care was also described in several of the included studies [40, 51], reflecting an awareness of the need for accessible abortion services as described by Shankar et al. [8]. Despite this, some participants still believed that abortion was best provided in private services by specialist abortion doctors [40, 42, 43, 44, 47]. Perceptions on the role of nurses and midwives in abortion care were similarly diverse [35, 40, 44], with varying support for them providing either direct or supportive abortion care. This may explain the challenges described by Mainey et al. [52] in implementing enhancements in nurse and midwife abortion scope of practice.

Experiences in promoting person‐centred care models for marginalised populations were described in several studies [34, 35, 39], as were perceptions of providers as client advocates [38, 39, 49]. Swedish doctors [53] experienced similar challenges caring for CALD abortion seekers as Singh et al. [35], noting the need for culturally appropriate approaches. Nurses and midwives in Pakistan [54] had to navigate similar barriers to the participants of Mainey's [38] 2023 study when supporting abortion seekers experiencing GBV. Nurses and midwives' perspectives presented in Qian et al. [3] affirmed a commitment to tailored quality care, mirroring the patient‐centred focus described in Malek's study [34]. Person‐centred care is universally perceived as essential to high‐quality abortion provider practice.

Australian abortion providers described experiencing multiple social and system‐based factors that affected abortion provision. Descriptions of clinicians having to navigate restrictive legislative frameworks to provide abortion care [45, 46, 50] add context to de Moel‐Mandel et al.'s [55] findings that restrictive abortion laws result in reduced abortion access for women. Challenges experienced when providing EMA in rural regional settings, particularly for vulnerable populations [43], give context to evidence of clients having to travel long distances for abortion care as [56, 57]. Frustration experienced by abortion providers in navigating these barriers is clearly articulated in these studies.

Participants in the included studies believed clinicians providing abortion should be highly skilled and have access to appropriate training at all levels [38, 39, 40, 42, 43, 44, 47]. Noting that the same question about whether abortion training should be included in the curriculum for RANZCOG Fellows and Trainees was applied in two surveys 10 years apart [42, 47] one wonders what progress has been made. Furthermore, recent findings from both medical students [55] and medical educators [58] indicate that abortion remains elusive in the medical curriculum. A lack of appropriate training reduces provider willingness and capacity to provide abortion [59, 60], which has implications for workforce competence and wellbeing into the future.

Articles published before decriminalisation convey a climate of fear for many clinicians seeking to provide abortion, with the risk of prosecution a clear consideration [45, 46, 50]. This fear was borne of genuine risk, with attempted prosecutions occurring within the pre‐decriminalisation context [61]. Provider distress related to the criminalisation of abortion provision is also described in United States (US) clinicians [1], who felt similar pressure to navigate legal risks. It is unclear what the legacy of the long period of criminalisation has been for the Australian clinicians who provided abortion during this time, noting the distress expressed by many in the review articles. Further research is needed into the impacts of these experiences, and how they have influenced how these abortion providers have adapted to the new legalised framework.

Review articles published after decriminalisation described different experiences related to dedicated abortion laws. Keogh et al. [23] explored clinicians' experiences of Victorian legislative conscientious objection provisions, while Bowman‐Smart et al. [37] and Haining et al. [22], investigated the experiences of clinicians involved in Victorian TRCs. While the number of studies investigating clinician perspectives in the post‐decriminalisation context was limited, they reflect the findings of Keogh et al. [10] that clinicians continue to experience challenges even after supportive abortion legislation is enacted. These few articles exploring the post‐decriminalised context highlight challenges with new legislation related to conscientious objection provisions; legislated requirements based on gestation; and the general complexities of implementing these regulations in practice, all of which would benefit from further scrutiny.

The review articles highlight the degree to which ethical challenges are central to abortion provision. The earliest review article focuses primarily on abortion decision‐making, and this theme remains though many of the included articles [22, 37, 40, 42, 47, 48]. Abortion care is provided along a gestational continuum, and the point at which this care aligns with an individual's principles of beneficence is not consistent [40, 42, 47]. Some clinicians in the included studies would prefer to limit abortion by gestation or medical indications [37, 40, 42, 47], preferences that are reflected in the wider literature [4, 62]. While some clinicians welcomed committees and frameworks for decision‐making, this was balanced by alternative views that these bodies/frameworks are overly restrictive [37]. This mirrors the experiences of similar abortion ethics committees in the USA [63]. The ethically challenging nature of abortion care only increases the complexity of abortion providers' practice.

The emotional effects of delivering abortion care, particularly at later gestations or by clinicians who hold an ethical ambivalence towards abortion, were important for some participants in the included studies [34, 40]. Multiple international studies have identified moral distress and burnout as a risk for some clinicians [4]. Reports from Malek et al.'s study that physically performing abortion procedures can cause distress also reflect similar findings in overseas studies [4, 24]. Negative emotional outcomes were often counterbalanced, however, by feelings of pride and job satisfaction in providing person‐centred care [2, 38, 49] and achieving positive outcomes for clients against the odds [38]. Understanding this complex interplay between negative and positive emotional responses to abortion provision is essential to ensuring provider wellbeing.

Experiences of abortion stigma were articulated in this scoping review [36, 39, 43, 47, 49]. Doctors in several included studies feared being perceived as the “abortion doctor” and sought to avoid or hide their abortion practice [34, 40]. This reflects similar findings from US providers [20] which found that 58% felt that disclosing their abortion practice is “not worth the hassle that could follow” p. 64. Some clinicians in the review studies perceived judgement from colleagues, family, and friends because of their abortion practice [44]. One participant spoke of fears of physical harm [49]. These negative emotional impacts are not confined to Australian providers, with multiple international studies [1, 2, 64] describing abortion providers feeling isolated and sometimes physically threatened because of abortion stigma.

Participants in several included studies experienced challenges related to conscientious objection [22, 23, 39, 43]. While most supported a clinician's right to conscientiously object [23], it is also clear that the practice results in negative outcomes for abortion seekers and abortion providers [1, 7]. Experiences with conscientious objection ranged from accommodating the appropriate exercise of legal provisions to witnessing misuse and abuse of the provisions to prevent abortions [23]. While not provided for under Australian legislation, institutional objection was also described by some participants, mirroring overseas experiences [23, 65]. The descriptions of conscientious objection align with the presence of low levels of conscientious objection expressed by the review article participants [40, 42, 47], with a small cohort of clinicians in the study populations identifying as being opposed to providing abortion care. A United Kingdom study identified similar accounts of nurses who were firmly opposed to abortion [66]. Considering that conscientious objection remains a persistent element of abortion care, the negative impact on abortion providers will likely be ongoing.

## 6 Conclusion

This review articulates the experiences and perceptions of abortion providers in Australia, and provides a foundation for future investment in research, education, and health policy. The paucity of recent research describing abortion providers' perceptions of abortion care education must be remediated to both assess current training options and ensure that any emerging training programmes provide a robust foundation for abortion provision. It is also important to explore the clinical, system‐based, emotional, and ethical challenges experienced by clinicians who are new to abortion provision or are delivering care in new settings. Targeted research into providers' experience of conscientious objection and abortion stigma could inform effective system‐based responses to minimise negative outcomes. Finally, it is essential to explore the respective impacts of decriminalisation and dedicated abortion legislation, to identify both the benefits of legislative change for clinicians as well as any emerging challenges in the decriminalised context.

Capturing the experiences and perceptions of abortion providers is critical to supporting the wellbeing of the abortion workforce. Contemporary experiential research can inform public health policy and health system changes to enhance support for both abortion seekers and clinicians. It is incumbent on legislators, regulators, and health service executives to listen to the experiences of providers practicing in the current context and take action to meet their needs. Effectively addressing the needs of the abortion workforce will better ensure the sustainability of abortion services into the future.

## 7 Limitations

- While care was taken in the developing search and selection criteria, including having no restrictions on the date or language of publication, it is unlikely but possible that relevant articles have been missed in the execution of the search.
- Articles containing Australian clinician experiences may have been excluded at the title and abstract screening stage if this experiential content was not clearly articulated as a focus of the study.
- There were articles excluded in the full‐text screening for which it was not possible to confirm that the participants included abortion providers. Any abortion providers' experiences in these excluded studies will not have been considered in this review.
- The search for this review was conducted in May 2024, meaning that potentially relevant articles published between the search and publication of this review will not have been considered.

## Funding

The authors have nothing to declare.

## Conflicts of Interest

Professor Caroline de Costa, one of the authors of this manuscript, is a Reviewer for the Australian and New Zealand Journal of Obstetrics and Gynaecology.

## Data Availability

The authors have nothing to report.

## Appendix 1 Data Extraction Summary

**Table:**

	Citation	State/territory	Aim/objectives	Study type	Participants/sample size	Findings
1	Malek M, Homer CS, McDonald C, Hannon CM, Moore P, Wilson AN. “Abortion Care at 20 Weeks and Over in Victoria: A Thematic Analysis of Healthcare Providers' Experiences,” *BMC Pregnancy and Childbirth*. 2024 Feb 6;24(1):112 [34]	Victoria	To examine health providers' perceptions and experiences of providing abortion care at 20 weeks and over for indications other than fetal or maternal medicine, as well as enablers and barriers to this care and how quality of care could be improved in one hospital in Victoria, Australia	Qualitative descriptive study using semi‐structured interviews	Doctors, nurses/midwives, social workers and other allied health professionals providing abortion care to women > 20 weeks gestation: 17 participants, including: – 5 medical officers – 7 nurses/midwives	*Key findings* – Staff commitment to unique, person‐centred care – System barriers cause delays and distress – Emotional overwhelm and ethical challenges – Perceived lack of services and challenges during COVID‐19 *Note:* Post‐decriminalisation in research jurisdiction.
2	Singh R, Mazza D, Moloney L, Shankar M, Subasinghe AK. “General Practitioner Experiences in Delivering Early Medical Abortion Services to Women From Culturally and Linguistically Diverse Backgrounds: A Qualitative–Descriptive Study,” *Age* (years). 2023 Aug 1;30(39):14 [35]	Victoria, Queensland, Tasmania, Western Australia (WA)	To: explore GP experiences in providing early medical abortion (EMA) services to women from CALD backgrounds; and describe GP perspectives on improving EMA care to this priority population in the Australian general practice setting	Qualitative descriptive study using semi‐structured interviews	General practitioners (GPs) who provide early medical abortion 18 participants	*Key findings*: – Challenges with communication and cultural competency – Impact of socio‐cultural gender roles and other influences on provision – Perception of abortion stigma and logistical access barriers to abortion provision – Importance of GP as a healthcare provider *Note:* post‐decriminalisation in all jurisdictions except WA.
3	Haas M, Church J, Street DJ, Bateson D, Mazza D. “How Can We Encourage the Provision of Early Medical Abortion in Primary Care? Results of a Best–Worst Scaling Survey,” *Australian Journal of Primary Health*. (2022) Dec 7 [36]	Australia – no breakdown by state/territory	From the perspective of GPs and Registered Nurses (RN)s working in Australia, what are the most important facilitators and barriers to the provision of EMA in primary care?	Descriptive, cross‐sectional study utilising the Best‐Worst Scaling (BWS), stated preference (SP) survey model	General Practitioners (GPs) and RNs 300 participants 150—GPs (13%) involved in abortion provision 150—RNs (16%) involved in abortion provision	*Key findings* – Key barriers for GPs include lack of clinical guidelines, clinical management and ability to provide follow‐up – Abortion stigma also impacted RNs – Communities of practice are considered protective/supportive *Note:* post‐decriminalisation in all jurisdictions except WA.
4	Haining CM, Bowman‐Smart H, O'Rourke A, de Crespigny L, Keogh LA, Savulescu J. “The ‘Institutional Lottery’: Institutional Variation in the Processes Involved in Accessing Late Abortion in Victoria, Australia.” In Women's Studies International Forum (2023) Nov 1 (Vol. 101, p. 102822). Pergamon [22]	Victoria	This paper reports on a subset of findings from a research project which examined Victorian Termination of Pregnancy Review Committees (TRCs) s that consider requests at later gestations Focusing on late term abortion processes that exist across institutions, the variation that exists and how this may impact the quality of care delivered	Qualitative descriptive study using semi‐structured interviews Same data as Bowman 2023	Health Professionals involved in TRCs 27 participants, including: Obstetrician/Maternal‐Fetal‐Medicine (MFM)—15 Psychiatrist—1 Neonatologist—1 Clinical geneticist—2 Genetic counsellor—6 Midwife—2	*Key findings* – Staff believe that the experience of clients accessing late abortion via a TRC varies significantly across institutions – The source of variation is perceived to be due to local regulations and institutional processes – Preference of fetal/medical grounds for abortion rather than social for abortion – Impacts of institutional conscientious objection are noted as are concerns about procedural fairness *Note:* Post‐decriminalisation in research jurisdiction.
5	Bowman‐Smart H, Keogh L, Haining CM, O'Rourke A, de Crespigny L, Savulescu J. “‘The Tabloid Test’: A Qualitative Interview Study on the Function and Purpose of Termination of Pregnancy Review Committees in Victoria, Australia,” *Reproductive Health*. (2023) Jul 18;20(1):104 [37]	Victoria	This study aimed to capture more details about the Victorian TRCs that consider requests at later gestations, including information about their purpose, decision‐making, and general processes The main focus of this paper is to provide insight into the purpose and function of TRCs	Qualitative descriptive study using semi‐structured interviews Same data as Haining 2023	Health Professionals involved in TRCs 27 participants, including: Obstetrician/Maternal‐Fetal‐Medicine (MFM)—15 Psychiatrist—1 Neonatologist—1 Clinical geneticist—2 Genetic counsellor—6 Midwife—2	*Key findings* – Participants noted the protective nature of TRCs for both staff and clients – Impact of external factors on processes/decision making (e.g., media/law) – Mitigation of reputational damage for institution and staff – Some perceptions that decisions about late abortion are often controversial and require frameworks – Some staff perceived the TRCs as gatekeeping and creating barriers *Note:* Post‐decriminalisation in research jurisdiction.
6	Mainey L, O'mullan C, Reid‐Searl K. “Working With or Against the System: Nurses' and Midwives' Process of Providing Abortion Care in the Context of Gender‐Based Violence in Australia.” *Journal of Advanced Nursing*. (2023) Apr;79(4):1329–41 [38]	Australia—no breakdown by state/territory	The aim of this study was to explain the process through which Australian nurses and midwives provide abortion care to people affected by Gender Based Violence (GBV)	Qualitative descriptive study using semi‐structured interviews Same data as Mainey 2022	Australian nurse (registered or enrolled) or midwife who had provided abortion care for at least 12 months with first‐hand experience of providing care to people victimised by GBV 18 participants	*Key findings* – All clinicians were committed to person‐centred care – Some participants perceived that they could work compliantly with the system to successfully provide abortion care – Other participants found themselves at odds with legislation, regulations, guidelines, settings, and care models, having to work outside the system to achieve outcomes – Clinician satisfaction with outcomes depended on the extend that they felt able to provide person‐centred care *Note:* post‐decriminalisation in all jurisdictions except WA.
7	Mainey L, O'Mullan C, Reid‐Searl K. “Unfit for Purpose: A Situational Analysis of Abortion Care and Gender‐Based Violence,” *Collegian*. (2022) Oct 1;29(5):557–65 [39]	Australia—no breakdown by state/territory	To explore how the broader situation affects the way nurses and midwives provide abortion care for victims of domestic violence or sexual assault	Qualitative descriptive study using semi‐structured interviews Same data as Mainey 2023	Australian nurse (registered or enrolled) or midwife who had provided abortion care for at least 12 months with first‐hand experience of providing care to people victimised by GBV 18 participants	*Key findings* – Participants found the healthcare system inadequate in supporting safe/appropriate abortion care, with skill‐mix and care pathway deficits noted – The socio‐legislative context negatively impacts clinicians and how care is provided – Education, training and system‐wide change is required to ensure that the system is fit—for‐purpose in providing abortion – System inadequacies seen as a cause of trauma for both staff and clients *Note:* Post‐decriminalisation in all jurisdictions except WA.
8	Desai A, Maier B, James‐McAlpine J, Prentice D, de Costa C. “Views and Practice of Abortion Among Queensland Midwives and Sexual Health Nurses,” *Australian and New Zealand Journal of Obstetrics and Gynaecology*. (2022) Apr;62(2):219–25 [40]	Queensland (post‐decriminal‐isation)	To examine the attitudes and practices of Registered Midwives and Sexual Health Nurses in Queensland towards induced abortion	Observational cross‐sectional survey design	Midwives and Sexual Health Nurses 623 participants (54% involved with abortion care)	*Key findings* – Over half of participants supported abortion in any situation – Participants indicated varying comfort with abortion depending on gestation and indication – Most were supportive of improved education and training for midwives/nurses – Fewer than half felt that providing abortion should be a part of nurse/midwife scope – A small group were completely opposed to abortion *Note:* Post‐decriminalisation in research jurisdiction.
9	Deb S, Subasinghe AK, Mazza D. “Providing Medical Abortion in General Practice: General Practitioner Insights and Tips for Future Providers,” *Australian Journal of General Practice*. (2020) Jun;49(6):331–7 [41]	Victoria (majority), New South Wales (NSW), Queensland, Northern Territory (NT), South Australia (SA)	To describe models of medical abortion care and to gain insights from current GP medical abortion providers as to how to establish and sustain medical abortion service delivery This article looks separately at models of care (MOC) and clinician perceptions	Qualitative descriptive study using semi‐structured interviews	GPs providing early medical abortion (EMA) 25 participants	*Key findings* – Three main models of care included: common, streamlined and ultrasonography‐inclusive – Some providers did not communicate their intention to provide abortion due to fears that their practice would restrict this activity – Preference for in‐clinic care and follow‐up – Importance of strong abortion provider networks – Only a small number perceived stigmatisation *Note:* Post‐decriminalisation in NSW, Vic & Queensland and pre‐decriminalisation in SA and NT.
10	Cheng HC, Black K, Woods C, de Costa C. “Views and Practices of Induced Abortion Among Australian Fellows and Trainees of The Royal Australian and New Zealand College of Obstetricians and Gynaecologists: A Second study,” *Australian and New Zealand Journal of Obstetrics and Gynaecology*. (2020) Apr;60(2):290–5 [42]	Australia—no breakdown by state.	To obtain an up‐to‐date understanding of the views and practices of members of the College regarding abortion and whether legislative changes had impacted these, by repeating the (2010) survey of RANZCOG members The goal of the 2010 survey was to conduct a survey of the opinions on and practice of abortion among Australian specialist obstetricians and gynaecologists	Observational cross‐sectional survey design	Australian RANZCOG Fellows and trainees 638 participants – 478 Fellows – 152 trainees – 2 not specified 74.5% perform abortions as a part of their practice	*Key findings* – 74.5% of obstetricians were involved in abortion requests and provision – 617 participants responded to the question of whether abortion should be part of general obstetrics/gynaecology practice, with 83.5% saying yes – 13.7% reported total opposition to abortion – Fewer than half who do not oppose abortion felt mifepristone had altered their practice – Other concerns related to the situations in which abortion was indicated, conflicting feelings, stigma, the need for clinical guidance, and client advocacy *Note:* Post‐decriminalisation in NSW, Vic & Queensland and pre‐decriminalisation in SA and NT.
11	Keogh LA, Gillam L, Bismark M, McNamee K, Webster A, Bayly C, Newton D. “Conscientious Objection to Abortion, the Law and Its Implementation in Victoria, Australia: Perspectives of Abortion Service Providers,” *BMC Medical Ethics*. (2019) Dec; 20:1–0 [23]	Victoria	Through the perspectives of experts on abortion services in Victoria, this study aimed to explore health professionals' understandings of the inclusion of Section 8 in the Abortion Law Reform Act, as well as their perceptions of how Section 8 has been implemented in the Victorian health system and its impact on care	Qualitative descriptive study using semi‐structured interviews Same data as Newton 2016	Abortion experts who are involved directly in the provision of either medical or surgical abortion, provided counselling in relation to accessing abortion, or are involved in policy or advocacy related to abortion access Total 19 participants – 14 either directly providing or working within a service that provided medical abortion – Included 13 medical officers and 2 nurses	All questions in this study related specifically to conscientious objection provisions in the Victorian abortion legislation *Key findings* – Participants were supportive of Section 8 as a mechanism to protect women's access – All participants had seen cases of these provisions being misused – Negative impacts of either use/misuse of conscientious objection provisions included guilt and discomfort for women, delay in access to abortion and rarely blocking of abortion access – Conscientious objection provisions were also perceived to reduce availability for providers and services as clinicians exercised them for various reasons *Note:* Post‐decriminalisation in research jurisdiction.
12	Dawson AJ, Nicolls R, Bateson D, Doab A, Estoesta J, Brassil A, Sullivan EA. “Medical Termination of Pregnancy in General Practice in Australia: A Descriptive‐Interpretive Qualitative Study,” *Reproductive Health*. (2017) Dec; 14:1–3 [43]	NSW	To explore the provision and referral of early medical termination of pregnancy (ToP) by GPs in NSW, Australia	Qualitative descriptive study using semi‐structured interviews and one focus group discussion	GPs and one GP/surgeon. 32 participants: – 8 abortion providers – 24 non‐providers	*Key findings* – Many participants believed that abortion is an essential service but do not want to be the abortion provider due to stigma – Perception that abortion care is quite specialised and requires specialised skills and setting – Abortion providers were cautious about advertising their work, and demand was variable – Need for better training opportunities and professional networks – Need for clear referral, escalation, and follow‐up pathways *Note:* Pre‐decriminalisation in research jurisdiction.
13	Newton D, Bayly C, McNamee K, Bismark M, Hardiman A, Webster A, Keogh L. “… a One Stop Shop in Their Own Community: Medical Abortion and the Role of General Practice,” *Australian and New Zealand Journal of Obstetrics and Gynaecology*. (2016) Dec;56(6):648–54 [44]	Victoria	To investigate the potential for expanding the role of general practice in the provision of EMA in Victoria by conducting interviews with current abortion providers and individuals with expertise in this area	Qualitative descriptive study using semi‐structured interviews Same data as Keogh 2019	Abortion experts who are involved directly in the provision of either medical or surgical abortion, provided counselling in relation to accessing abortion, or are involved in policy or advocacy related to abortion access Total 19 participants – 14 either directly providing or working within a service that provided medical abortion – Included 13 medical officers and 2 nurses	*Key findings* – Participants were very supportive of EMA provision in GP – Primary care provision would increase access and possibly improve workforce shortages – Nurse involvement in multidisciplinary care was mostly supported – Some views that abortion might be best provided in specialist services – Education and training needs and provider partnerships would be essential to developing services *Note:* Post‐decriminalisation in research jurisdiction.
14	Black KI, Douglas H, de Costa C. “Women's Access to Abortion After 20 weeks' Gestation for Fetal Chromosomal Abnormalities: Views and Experiences of Doctors in New South Wales and Queensland,” *Australian and New Zealand Journal of Obstetrics and Gynaecology*. (2015) Apr;55(2):144–8 [45]	Queensland and NSW	To explore the legal and practical issues identified by abortion providers in NSW and Queensland when an abortion is requested beyond 20‐weeks' gestation The article also describes the role hospital ethics committees play in the process for women whose fetus has been diagnosed with an abnormality	Qualitative descriptive study using semi‐structured interviews Same data as Douglas 2013 and de Costa 2013	Medical doctors who provide abortion services 22 participants: – 4 MFMs – 4 Sexual Health Physicians – 10 private abortion providers – 3 Obstetrician/Gynaecologists – 1 family planning doctor (did not perform abortion)	Participants were asked to review the law in their state and then consider three scenarios of abortion requests related to fetal anomaly > 20 weeks of gestation. *Key findings* – Most participants believed that complicated laws and health policies contributed to complex and restricted access to abortion after 20 weeks – Ethics committees were perceived to cause delays and uncertainty as to outcome – Uncertainty and delays caused stress for both women and abortion providers, often resulting in referrals for abortion interstate *Note:* Pre‐decriminalisation in research jurisdictions.
15	Baird B. “‘Happy Abortionists’ Considering the Place of Doctors in the Practice of Abortion in Australia Since the Early 1990s,” *Australian Feminist Studies*. (2014) Oct 2;29(82):419–34 [49]	SA, Queensland, Victoria and Tasmania	To amplify the subjective and institutional negotiations that have facilitated, indeed in some cases improved, abortion services over the previous 20 years	Qualitative descriptive study using semi‐structured interviews	Not clear if it is two separate interview studies OR one study with a subgroup analysis: – 15 participants in first reference to interviews (Not all provide abortion) – Sub‐group: 4 doctors who have provided abortion across the four states over the last 20 years	*Key findings* – Participants noted harm minimisation and preventing negative outcomes of illegal abortions – Perception that women accessing abortion are not aware of the history nor challenges of doctors providing abortion – Concerns around stigma and violence towards abortion providers – Awareness of allies in abortion provision and general job satisfaction *Note:* Pre‐decriminalisation in research jurisdictions, except Victoria.
16	de Costa C, Douglas H, Black K. “Making It Legal: Abortion Providers' Knowledge and Use of Abortion Law in New South Wales and Queensland,” *Australian and New Zealand Journal of Obstetrics and Gynaecology*. (2013) Apr;53(2):184–9 [46]	NSW & Queensland	To explore the knowledge in regard to abortion law of doctors providing abortion in NSW and Queensland, and their views on this law, and seeking their descriptions of how they applied that knowledge to their day‐to‐day practice Participants were asked to review the law in their state and then consider 10 separate scenarios and asked to comment on the legality and availability	Qualitative descriptive study using semi‐structured interviews Same data as Black 2015 and Douglas 2013	Medical doctors who provide abortion services 22 participants: – 4 MFMs – 4 Sexual Health Physicians – 10 private abortion providers – 3 obstetrician/gynaecologists – 1 family planning doctor (did not perform abortion)	*Key findings* – Participants were aware that abortion was a crime in their state at time of study – All were aware of need to ensure that assessment, decision and documentation conformed to these laws – All felt that these restrictions were unnecessary and should be changed – Most felt equipped to assess emotional distress themselves – Many were welcoming of law reform and found the existing processes demeaning for women *Note:* Pre‐decriminalisation in research jurisdictions.
17	Douglas H. “Manufacturing Mental Illness (and Lawful Abortion): Doctors' Attitudes to Abortion Law and Practice in New South Wales and Queensland,” H Douglas, K Black, C de Costa, “Manufacturing Mental Illness (and Lawful Abortion): Doctors' Attitudes to Abortion Law and Practice in New South Wales and Queensland” (2013). 2013;20:560–76 [50]	NSW & Queensland	To investigate the current understanding (of abortion law) of doctors who provide abortion services to women This article focuses on how doctors interpreted the phrase “preservation of the mother's life” in the context of two scenarios and how this affects practice and participants were asked to review the law in their state and then consider 2 of 10 separate scenarios and asked to comment on the legality and availability	Qualitative descriptive study using semi‐structured interviews Same data as Black 2015 and de Costa 2013	Medical doctors who provide abortion services 22 participants: – 4 MFMs – 4 Sexual Health Physicians – 10 private abortion providers – 3 obstetrician/gynaecologists – 1 family planning doctor (did not perform abortion)	*Key findings* – Participants demonstrated how they might need to assess each case in the context of the legislation – Participants felt compelled to behave misleadingly, dishonestly and often unethically to provide a “legal” abortion – Doctors focused on mental health concerns to justify abortion – Participants felt frustrated at having to behave this way to support their client's request for abortion, but noted genuine risks of prosecution *Note:* Pre‐decriminalisation in research jurisdictions.
18	de Costa CM, Russell DB, Carrette M. “Views and Practices of Induced Abortion Among Australian Fellows and Specialist Trainees of the Royal Australian and New Zealand College of Obstetricians and Gynaecologists,” *Medical Journal of Australia*. 2010 Jul;193(1):13–6 [47]	Australia – no breakdown by state.	To conduct a survey of the opinions on and practice of abortion among Australian specialist obstetricians and gynaecologists	Observational cross‐sectional survey design	Australian RANZCOG Fellows and trainees 740 participants – 583 fellows – 142 trainees – 15 not specified 73% perform abortion as a part of their practice	*Key findings* – 14.6% opposed abortion completely – Of 632 participants who did not completely oppose abortion, 81.8% believed abortion should be a part of routine practice – Abortion was usual practice for 73.3% (463) of those not opposed to abortion although 204 of the 463 (44%) only provide it for significant fetal anomaly – 15 respondents in small communities did not provide abortion as they did not want to become the “abortion doctor” – Over 50% of those not opposed to abortion believed that Mifepristone would alter their practice *Note:* Pre‐decriminalisation in research jurisdictions except Victoria.
19	Petersen KA. “Abortion Counselling in Australia,” *Australian Journal of Sex*, *Marriage and Family*. (1985) May 1;6(2):93–103 [48]	All Australian capital cities	To examine the abortion delivery system in Australia to assess the availability of and access to abortion services, and the relationship between the laws on and practice of abortion Respondents were asked if they favoured the introduction of compulsory counselling for women seeking abortions	Observational cross‐sectional survey design Some semi‐structured interviews	Doctors, nurses, counsellors and social workers All doctors in the sample either performed abortions or referred for abortion. 176 participants	This article focused solely on clinician's views on compulsory pre‐abortion counselling. *Key findings* – Most respondents were in favour of a women's right to choose to have an abortion or continue the pregnancy – 66.3% (114) favoured compulsory counselling – All disciplines surveyed had similar views on counselling – More clinicians in Adelaide and Darwin favoured compulsory counselling pre‐ abortion – Melbourne respondents were less supportive of compulsory counselling – Main reasons for not advising or approving abortion were: maternal ambivalence; pressure from the partner or family, advanced pregnancy *Note:* Pre‐decriminalisation in research jurisdictions.
